# Tryptophan Catabolism as Immune Mechanism of Primary Resistance to Anti-PD-1

**DOI:** 10.3389/fimmu.2020.01243

**Published:** 2020-07-07

**Authors:** Andrea Botticelli, Silvia Mezi, Giulia Pomati, Bruna Cerbelli, Edoardo Cerbelli, Michela Roberto, Raffaele Giusti, Alessio Cortellini, Luana Lionetto, Simone Scagnoli, Ilaria Grazia Zizzari, Marianna Nuti, Maurizio Simmaco, Paolo Marchetti

**Affiliations:** ^1^Department of Clinical and Molecular Medicine, Sapienza University of Rome, Rome, Italy; ^2^Department of Radiological, Oncological and Pathological Sciences, Faculty of Medicine and Dentistry, Sapienza University of Rome, Rome, Italy; ^3^Medical Oncology Unit, Sant'Andrea Hospital of Rome, Rome, Italy; ^4^Medical Oncology Unit, San Salvatore Hospital, L'Aquila, Italy; ^5^Experimental Immunology Laboratory, Biochemistry Laboratory, IDI-IRCCS FLMM, Rome, Italy; ^6^Department of Medical and Surgical Sciences and Translational Medicine, Sapienza University of Rome, Rome, Italy; ^7^Department of Experimental Medicine, Faculty of Medicine and Dentistry, Sapienza University of Rome, Rome, Italy; ^8^Advanced Molecular Diagnostics Unit, Sant'Andrea Hospital, Sapienza University of Rome, Rome, Italy

**Keywords:** indoleamine-2,3-dioxygenase, tryptophan metabolism, tumor immunity, kynurenine, anti-PD-1

## Abstract

**Background:** Clinical trials showed that only a subset of patients benefits from immunotherapy, suggesting the need to identify new predictive biomarker of resistance. Indoleamine-2,3-dioxygenase (IDO) has been proposed as a mechanism of resistance to anti-PD-1 treatment, and serum kynurenine/tryptophan (kyn/trp) ratio represents a possible marker of IDO activity.

**Methods:** Metastatic non-small cell lung cancer (NSCLC), renal cell carcinoma (RCC), and head and neck squamous cell carcinoma (HNSCC) treated with nivolumab as second-line treatment were included in this prospective study. Baseline serum kyn and trp levels were measured by high-performance liquid chromatography to define the kyn/trp ratio. The χ^2^-test and *t*-test were applied to compare frequencies and mean values of kyn/trp ratio between subgroups with distinct clinical/pathological features, respectively. Median baseline kyn/trp ratio was defined and used as cutoff in order to stratify the patients. The association between kyn/trp ratio, clinical/pathological characteristics, response, progression-free survival (PFS), and overall survival (OS) was analyzed.

**Results:** Fifty-five patients were included. Mean baseline serum kyn/trp ratio was significantly lower in female than in male patients (0.048 vs. 0.059, respectively, *p* = 0.044) and in patients with lung metastasis than in others (0.053 vs. 0.080, respectively, *p* = 0.017). Mean baseline serum kyn/trp ratio was significantly higher in early progressor patients with both squamous and non-squamous NSCLC (*p* = 0.003) and with a squamous histology cancer (19 squamous NSCLC and 14 HNSCC, *p* = 0.029). The median value of kyn/trp ratio was 0.06 in the overall population. With the use of median value as cutoff, patients with kyn/trp ratio > 0.06 had a higher risk to develop an early progression (within 3 months) to nivolumab with a trend toward significance (*p* = 0.064 at multivariate analysis). Patients presenting a baseline kyn/trp ratio ≤0.06 showed a longer PFS [median 8 vs. 3 months; hazard ratio (HR): 0.49; 95% confidence interval (CI) 0.24–1.02; *p* = 0.058] and a significantly better OS than did those with a kyn/trp ratio > 0.06 (median 16 vs. 4 months; HR: 0.39; 95% CI 0.19–0.82; *p* = 0.013).

**Conclusion:** Serum kyn/trp ratio could have both prognostic and predictive values in patients with solid tumor treated with immunotherapy, probably reflecting a primary immune-resistant mechanism regardless of the primary tumor histology. Its relative weight is significantly related to gender, site of metastasis, NSCLC, and squamous histology, although these suggestive data need to be confirmed in larger studies.

## Introduction

Immune checkpoint inhibitors (ICIs), a class of drugs able to block immunosuppressive pathways in order to prime an anticancer immunity, revolutionized the standard of care in many solid tumors, including non-small cell lung cancer (NSCLC), recurrent/metastatic head and neck squamous cell carcinoma (R/M-HNSCC), and renal cell carcinoma (RCC) (1). In NSCLC, the programmed cell death protein 1 (PD-1) inhibitor, nivolumab showed long-term benefit in a significant proportion of pretreated patients with a 2-years overall survival (OS) of 23% and 29% in squamous and non-squamous histology, respectively, over-performing standard chemotherapy (1, 2). Nevertheless, emerging data from clinical trials showed that only 20–25% of pretreated patients with NSCLC really benefit from immunotherapy with ICI monotherapy (3–5). Recently, nivolumab and the anti-PD-1 pembrolizumab showed a significant activity in patients with HNSCC who progressed on or after platinum-based regimens (6, 7); consequently, both the drugs have been approved by Food and Drug Administration (FDA) for platinum-refractory R/M-HNSCC. However, CheckMate-141 (6) failed to demonstrate a significant association between PD-L1 expression, using different thresholds of expression in tumor cells, response rates to the anti-PD-1 nivolumab and OS not allowing any selection of patients eligible for treatment.

In RCC, nivolumab entered in clinical practice on the basis of the results of a phase III study that demonstrated an advantage in OS after first-line treatment with tyrosine-kinase inhibitors (TKIs) (8). Nivolumab was approved based on the CheckMate 025, demonstrating the superiority of nivolumab compared with everolimus in terms of OS but not progression-free survival (PFS). In the near future, immunotherapy will change the first-line standard of care of metastatic RCC on the basis of the results from a more recent phase III trial evaluating the combination of nivolumab and the anti-CTLA-4 monoclonal antibody ipilimumab (9). Actually, PD-L1 expression seems to have a prognostic value in RCC, but it is far from being considered as a marker of treatment benefit.

The emerging data from these clinical trials showed that only a relatively small subset of patients really benefit from ICIs, underlining the crucial role of patient selection in the choice of the best therapeutic strategy. Although PD-L1 expression in tumor microenvironment has been explored in several retrospective and prospective clinical trials, across many different tumor types, all the results suggest caution in considering PD-L1 as a reliable method for the selection of eligible patients for immunotherapy (4, 10–14). The expression of PD-L1 is dynamic and is the result of complex molecular crosstalk between different intracellular pathways, such as MAPK, PI3K, and Aky/PKB (15, 16). Although some other biomarkers of response to ICI have been proposed, such as tumor mutational burden and mismatch repair gene defect, biomarkers of primary resistance to immunotherapy are still lacking (17), and the mechanisms of immunoresistance in many types of cancer still remain largely unknown and poorly predictable before starting immunotherapy (18). The essential amino acid tryptophan (trp) catabolism is recognized as an important microenvironmental factor that suppresses antitumor immune responses in cancer. Depletion of trp, a fundamental factor for T-cell metabolism, is one of the main mechanisms involved in primary resistance to immunotherapy leading to T-cell anergy and apoptosis (19). Indoleamine-2,3-dioxygenase (IDO) is an enzyme able to catalyze the first and rate-limiting reaction of the essential amino acid l-tryptophan (trp) conversion into l-kynurenine (kyn), inducing an immunosuppressive microenvironment in cancers (20). IDO activity is involved in peripheral immune tolerance because it can promote the inhibition of T-cell proliferation induced by trp deprivation (19, 21–25). Moreover, IDO activity could represent the central and immunobiologically relevant enzyme of tumor immune escape, and it could be involved in the development of primary resistance to treatment with ICI (26).

In a previous study, a high level of kyn that cooperates with trp in suppression of antitumor immune-response by inducing regulatory T cells (Treg) (27) has been shown to correlate with advanced stage at diagnosis, worse prognosis, and response to chemotherapy (28–30). A crucial role in Treg expansion is determined by myeloid-derived suppressor cell (MDSC), which represents a myeloid cell population, at different grades of differentiation, involved in the inhibition of both the innate and adaptive immune response favored by the IDO activity. MDSCs are involved in the Treg expansion through an IDO-mediated mechanism. Indeed, the interaction between MDSC and activated T cell promotes the conversion of effector T cell in Treg (31, 32). Moreover, kyn promotes or suppresses neoplastic transformation and tumorigenesis and drives tumor growth in autocrine fashion, inducing survival and cell motility as described in malignant glioma cells (27). In addition to IDO, alternative enzymatic pathways of trp catabolism involving tryptophan-2,3-dioxygenase (TDO) (33) and with lesser extent IDO2 (34–36) may also be involved in trp metabolism contributing to immune escape mechanism and tumor progression. IDO activity could represent one of the biomarkers of resistance to immunotherapy as universal and agnostic, not linked to individual neoplasms, easily assessable, and virtually useful in treatment planning and in the correct selection of cancer patients for immunotherapy.

The objective of this study was to investigate the possible association between the serum baseline kyn/trp ratio and the response to immunotherapy in patients affected by NSCLC, RCC, and R/M-HNSCC.

## Methods and Materials

### Patient Population

Patients eligible for second-line treatment with nivolumab with metastatic RCC, who progressed after first line with the TKIs pazopanib or sunitinib, as well as patients with metastatic NSCLC non-oncogene addicted, progressed after first-line chemotherapy as well as recurrent/metastatic platinum refractory HNSCC, followed up at Policlinico Umberto I and at Policlinico Sant'Andrea, in Rome, from June 2016 to May 2019, were enrolled into this prospective study. Eligible patients were those aged >18 years with an Eastern Cooperative Oncology Group (ECOG) performance status ≤ 2 and adequate cardiac, pulmonary, renal, liver, and bone marrow function. Inclusion criteria were histologically confirmed diagnosis of RCC, NSCLC, and HNSCC; measurable disease according to RECIST version 1.1; and written informed consent. Exclusion criteria were autoimmune disease; symptomatic interstitial lung disease and any other significant comorbidity; systemic pharmacological immunosuppression; prior treatment with immune-stimulatory antitumor agents including checkpoint-targeted agents; patients who received checkpoint inhibitor in other setting; and patients with gastrointestinal malabsorption disorders that could modify the serum level of trp.

Nivolumab treatment was administered in NSCLC and in RCC at the dose of 3 mg/kg every 2 weeks i.v. until disease progression or development of unacceptable toxicity. Patients with HNSCC received 240 mg i.v. flat dose every 2 weeks until disease progression or unacceptable toxicity. Radiological response was assessed with i-RECIST Criteria and classified according to disease control (complete response, partial response, and stable disease) and progressive disease. Patients experiencing disease progression within 3 months from the beginning of nivolumab were defined as early progressors. All toxicity was graded according to the National Cancer Institute Common Terminology Criteria for Adverse Events (version 4.0), and toxicity assessments were performed at day 1 of every cycle until the end of treatment. PFS was defined as the time from patient registration on this prospective study until the first documented tumor progression or death from any cause. OS was defined as the time from patient registration to death from any cause. The association between kyn/trp ratio, clinical/pathological characteristics (including the analysis by tumor sites as well as by histology), response, PFS, and OS was analyzed. The study was conducted in accordance with good clinical practice guidelines and the Declaration of Helsinki. The study protocol and the final version of the protocol were approved by the Institutional Ethics Committee (CE 4421).

### Tryptophan and Kynurenine Quantifying Analysis

We evaluated serum levels of trp and kyn by a modified liquid chromatography–tandem mass spectrometry (LC–MS/MS) method. Serum samples were collected and stored at −80°C until analysis. Fifty microliters of serum samples was deproteinized using 50 μl of internal standard (IS) solution [50 μM in tricarboxylic acid (TCA) 4%], vortex mixed, and centrifuged at 14,000 rpm for 15 min. Twenty microliters of clean upper layer was injected into a chromatographic system. Chromatographic separation of analytes was performed using an Agilent Liquid Chromatography System series 1100 (Agilent Technologies, USA), on a biphenyl column (100 × 2.1 mm, Kinetex 2.6 μm of biphenyl, 100 Å, Phenomenex, CA, USA) equipped with a security guard precolumn (Phenomenex, Torrance, CA, USA). The mobile phase consisted of a solution of 0.1% aqueous formic acid (A) and 100% methanol (B); elution was performed at flow rate of 400 μl/min, using an elution gradient. The MS method was performed on a 3200 triple quadrupole system (Applied Biosystems, Foster City, CA, USA) equipped with a Turbo Ion Spray source, as previous described (37). The detector was set in the positive ion mode. The instrument was set in the multiple reaction monitoring (MRM) mode. Data were acquired and processed by the Analyst 1.5.1 Software. A threshold to identify an unfavorable ratio was defined as >0.06, derived from the median kyn/trp ratio detected in the overall population, because a conventional cutoff has not yet been established.

### Statistical Analysis

In the descriptive analysis, quantitative variables were described as mean and range, whereas qualitative variables as number and percentage. The χ^2^-test and *t*-test for unpaired data were applied to compare frequencies and means, respectively. PFS and OS were estimated using the Kaplan–Meier method, comparisons between groups were made using the log-rank test, and the Mantel–Cox method was used to generate hazard ratios (HRs) and 95% confidence intervals (CIs). Comparison was evaluated using the non-parametric Mann–Whitney *U* test. To identify factors associated with early progressors, univariate and multivariate logistic regression models were used. According to the kyn/trp cutoff value of 0.06, we used kyn/trp ratio as a dichotomous variable for the analyses (kyn/trp ratio > 0.06 vs. kyn/trp ratio ≤ 0.06). The results of univariate and multivariate analyses were expressed in odds ratio and 95% CIs. Statistical significance was set at *p* < 0.05. Statistical analysis was performed using IBM SPSS Statistics Version 24.0 (Armonk, NY, USA).

## Results

### Clinical Characteristics

**Figure 1:**
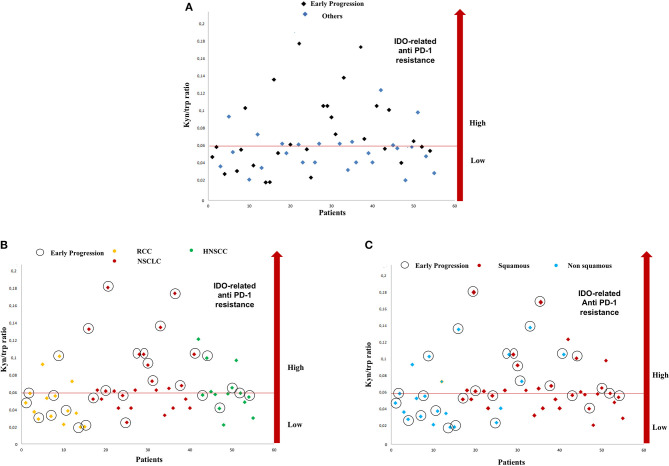
Distribution of serum kyn/trp ratio and early progressions in study population **(A)**. Distribution of serum kyn/trp ratio and early progression according to primary tumor site **(B)** and on the basis of the squamous histology **(C)**. kyn, kynurenine; trp, tryptophan.

Fifty-five metastatic patients treated with nivolumab were enrolled in this study: 26 patients in the NSCLC group, 15 patients in the RCC group, and 14 patients in the HNSCC group. Baseline clinical–pathological characteristics of patients are summarized in Table 1. Among lung cancer patients, 19 patients had squamous cell carcinoma, whereas the remaining had non-squamous histology (six adenocarcinoma and one undifferentiated tumor). All 15 patients in the RCC group had clear cell carcinoma histology. Thirty-nine patients were male (70.9%), 16 patients were female (29.1), and median age was 65 years (range 44–85). All patients were assessed at baseline for serum trp and kyn levels. The median value of kyn/trp in the overall population was 0.06 (range 0.018–0.180) (Figure 1).

**Table 1:** Association between baseline clinicopathological characteristics of the study population and kyn/trp ratio.

**Characteristics**	***N* (%)**	**Median (range)**	**Serum kyn/trp ratio (mean ± SD)**	***P*-value**
**AGE**		65 (44–85)		
>65	31 (56.4)		0.056 ± 0.038	0.121
<65	24 (43.6)		0.048 ± 0.029	
**BMI**		22.4 (16.9–37.5)		
Normal	39 (70.9)		0.058 ± 0.031	0.839
Overweight/obese	13 (23.6)		0.054 ± 0.040	
Underweight	3 (5.5)		0.056 ± 0.025	
**SEX**				
Male	39 (70.9)		0.059 ± 0.037	0.044*
Female	16 (29.1)		0.048 ± 0.020	
**HISTOLOGY**				
Clear cell carcinoma	15 (27.3)		0.036 ± 0.024	0.054
Squamous NSCLC	19 (34.5)		0.060 ± 0.04	
Adenocarcinoma	6 (10.9)		0.100 ± 0.04	
Undifferentiated NSCLC	1 (1.8)		0.040	
Squamous HNSCC	14 (25.5)		0.055 ± 0.034	
**BASELINE (ECOG) PS**				
PS 0	38 (69.1)		0.053 ± 0.025	0.054
PS 1	17 (30.9)		0.056 ± 0.047	
**BRAIN METASTASIS**				
Yes	6 (10.9)		0.064 ± 0.034	0.905
No	47 (89.1)		0.055 ± 0.035	
**LUNG METASTASIS**				
Yes	40 (72.7)		0.053 ± 0.029	0.017*
No	15 (27.2)		0.080 ± 0.046	
**PLEURAL EFFUSION**				
Yes	6 (10.9)		0.062 ± 0.051	0.096
No	49 (89.1)		0.054 ± 0.032	
**LIVER METASTASIS**				
Yes	9 (16.4)		0.062 ± 0.050	0.076
No	46 (83.6)		0.054 ± 0.030	
**EARLY PROGRESSOR**				
Yes	29 (52.7)		0.056 ± 0.042	0.047*
No	26 (47.3)		0.050 ± 0.021	

kyn, kynurenine; trp, tryptophan; SD, standard deviation; BMI, body mass index; NSCLC, non-small cell lung cancer; HNSCC, head and neck squamous cell carcinoma; ECOG PS, Eastern Cooperative Oncology Group performance status; *Statistical significance was set at p < 0.05.

### Association Between Serum Kynurenine/Tryptophan Ratio, Clinicopathological Features, and Response to Immunotherapy

The association between mean baseline kyn/trp ratio and clinicopathological characteristics was analyzed as shown in Table 1.

Mean serum kyn/trp ratio was significantly lower in female than in male patients (0.048 vs. 0.059, respectively, *p* = 0.044). Moreover, in patients with lung metastasis, mean serum kyn/trp ratio was 0.053 vs. 0.080 in other patients (*p* = 0.017). No significant association was found between baseline serum kyn/trp ratio and age, body mass index (BMI), histology, baseline ECOG PS, or the presence of metastasis in the brain, liver, and pleura (Table 1).

**Figure 2:**
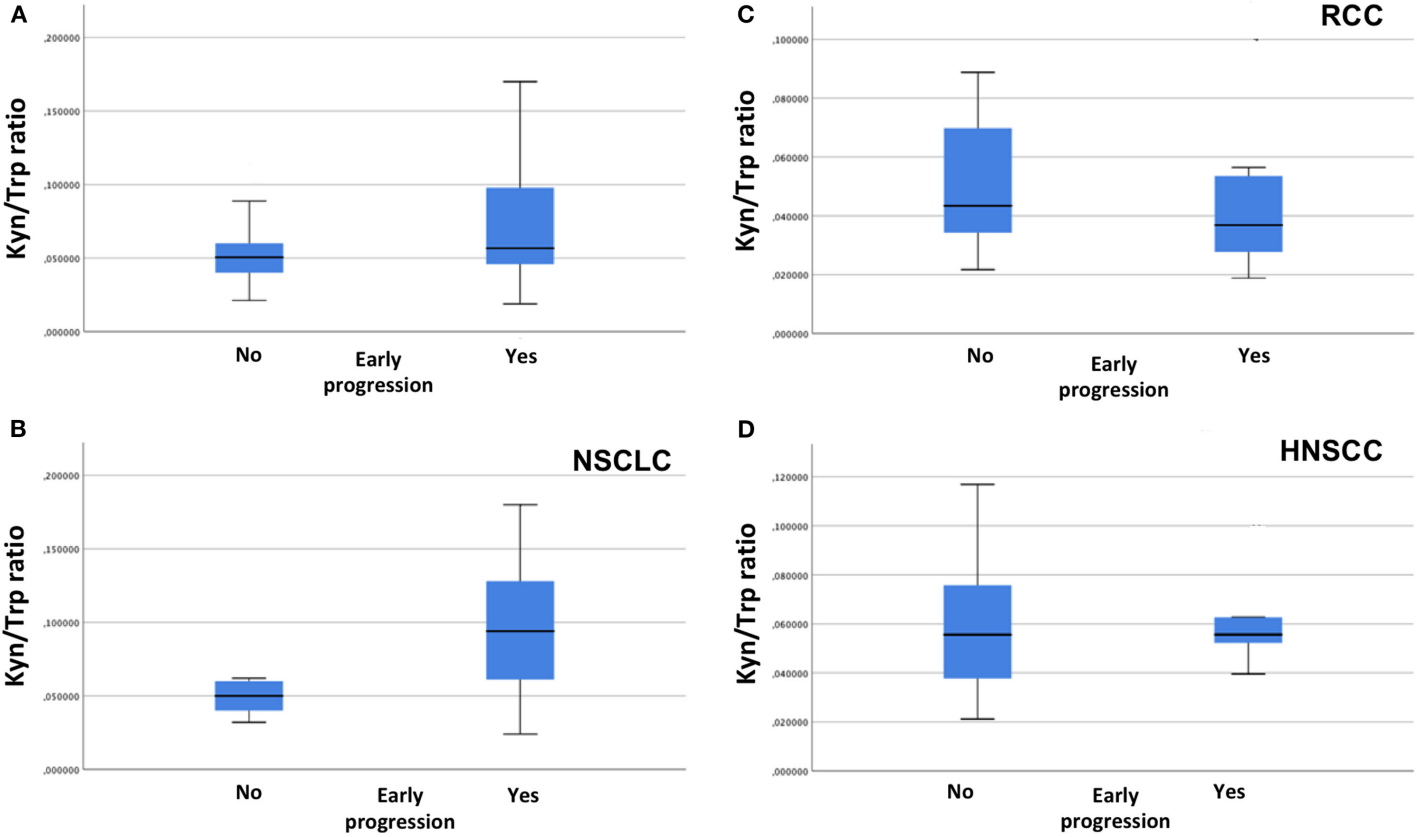
Association between serum kyn/trp ratio and early progression in overall population **(A)**, in NSCLC group **(B)**, in RCC group **(C)**, and in HNSCC group **(D)** (Mann–Whitney *U* test). kyn, kynurenine; trp, tryptophan; NSCLC, non-small cell lung cancer; RCC, renal cell carcinoma; HNSCC, head and neck squamous cell carcinoma.

With a median follow-up of 7.75 months, 11 (20%), 13 (23.6%), and 31 (56.3%) patients had a stable disease (SD), a partial response (PR), and a progressive disease (PD), respectively. An early progression (within 3 months from the start of immunotherapy) occurred in 29 patients (52.7%). The distribution of early progression in the study population is shown in Figure 1, according to the serum kyn/trp ratio (Figure 1A), primary tumor site (Figure 1B) and on the basis of the analysis by histology, the squamous one (Figure 1C). Overall, patients who showed an early progression had a slightly but significantly higher mean kyn/trp ratio than had others (0.056 vs. 0.050, respectively, *p* = 0.047) (Table 1 and Figure 2A). In patients with NSCLC, regardless of the different histotypes, mean serum kyn/trp ratio was significantly higher in early progressors (0.094 vs. 0.050; *p*-value = 0.003), as shown in Table 2 and Figure 2B, whereas no significant association was found between kyn/trp ratio and early progression in the RCC and HNSCC groups (Table 2 and Figures 2C,D). Nevertheless, in the RCC group, there seems to be a tendency to an inverse correlation between kyn/trp ratio and early progression (Figure 2C). Considering all patients with squamous histology (both squamous NSCLC and HNSCC), mean kyn/trp ratio was higher in early progressors than in patients who experienced initial benefit from immunotherapy (0.072 vs. 0.055, respectively; *p*-value = 0.029, Table 2).

**Table 2:** Association between kyn/trp ratio, early progression, and primary tumor.

	***N* (%)**	**Kyn/trp mean (±SD)**	***P*-value**
RCC	15	0.036 ± 0.024	
PD < 3 months (yes vs. no)	9 (60.0) vs. 6 (40.0)	0.036 vs. 0.043	0.590
NSCLC	26	0.06 ± 0.040	
PD < 3 months (yes vs. no)	14 (53.8) vs. 12 (46.2)	0.094 vs. 0.050	0.003*
HNSCC	14	0.055 ± 0.026	
PD < 3 months (yes vs. no)	6 (42.8) vs. 8 (57.2)	0.055 vs. 0.055	0.961
Squamous histology (NSCLC and HNSCC)	33	0.057 ± 0.035	
PD < 3 months (yes vs. no)	14 (42.4) vs. 19 (57.6)	0.072 vs. 0.055	0.029*

kyn, kynurenine; trp, tryptophan; SD, standard deviation; RCC, renal cell carcinoma; PD, progressive disease; NSCLC, non-small cell lung cancer; HNSCC, head and neck squamous cell carcinoma; *Statistical significance was set at p < 0.05.

With the use of a univariate analysis (Table 3), age, PS ECOG 1, and a baseline kyn/trp ratio higher than the median value (>0.06) were significantly associated with an early progression of disease.

**Table 3:** Univariate and multivariate analyses: association between patients characteristic and early progression.

	**Univariate analysis**		**Multivariate analysis**	
	**OR (95% CI)**	***P***	**OR (95% CI)**	***P***
Age (>65 vs. <65)	3.32 (104–10.58)	0.042*	2.71 (0.74–9.97)	0.132
BMI (overweight vs. other)	1.33 (0.36–4.92)	0.666	–	
Sex (female vs. male)	0.85 (0.26–2.74)	0.795	–	
Histology (sq vs. non-sq)	0.34 (0.11–1.06)	0.065	–	
ECOG (PS 1 vs. PS 0)	7.15 (1.75–29.20)	0.006*	6.80 (1.45–31.74)	0.015*
Brain metastasis (yes vs. no)	1.76 (0.29–10.55)	0.536	–	
Lung metastasis (yes vs. no)	0.30 (0.07–1.28)	0.104	–	
Pleural effusion (yes vs. no)	0.80 (0.14–4.42)	0.806	–	
Liver metastasis (yes vs. no)	3.5 (0.65–18.75)	0.144	–	
Kyn/trp median (≤0.06 vs. >0.06)	0.25 (0.07–0.86)	0.028*	0.24 (0.05–1.08)	0.064*

*OR, odds ratio; BMI, body mass index; Sq, squamous; ECOG, Eastern Cooperative Oncology Group; PS, performance status*;

* *Statistical significance was set at p < 0.05*.

With the use of a multivariate analysis, including age, kyn/trp ratio, and PS, only PS was still significantly associated with early progression (*p* = 0.015, Table 3), whereas the association between a kyn/trp ratio > 0.06 and early progression was not confirmed in the overall population (*p* = 0.064).

### The Serum Kynurenine/Tryptophan Ratio and Clinical Outcomes

Median PFS was 4 months (range 1–28 months) and median OS (range 1–28) was 5 months in the whole cohort.

**Figure 3:**
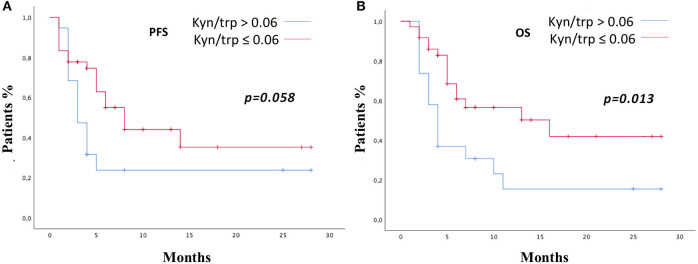
PFS **(A)** and OS **(B)** in the study population according to kyn/trp ratio >0.06 or ≤0.06 were addressed by the Kaplan–Meier method and log-rank test. PFS, progression-free survival; OS, overall survival; kyn, kynurenine; trp, tryptophan.

**Figure 4:**
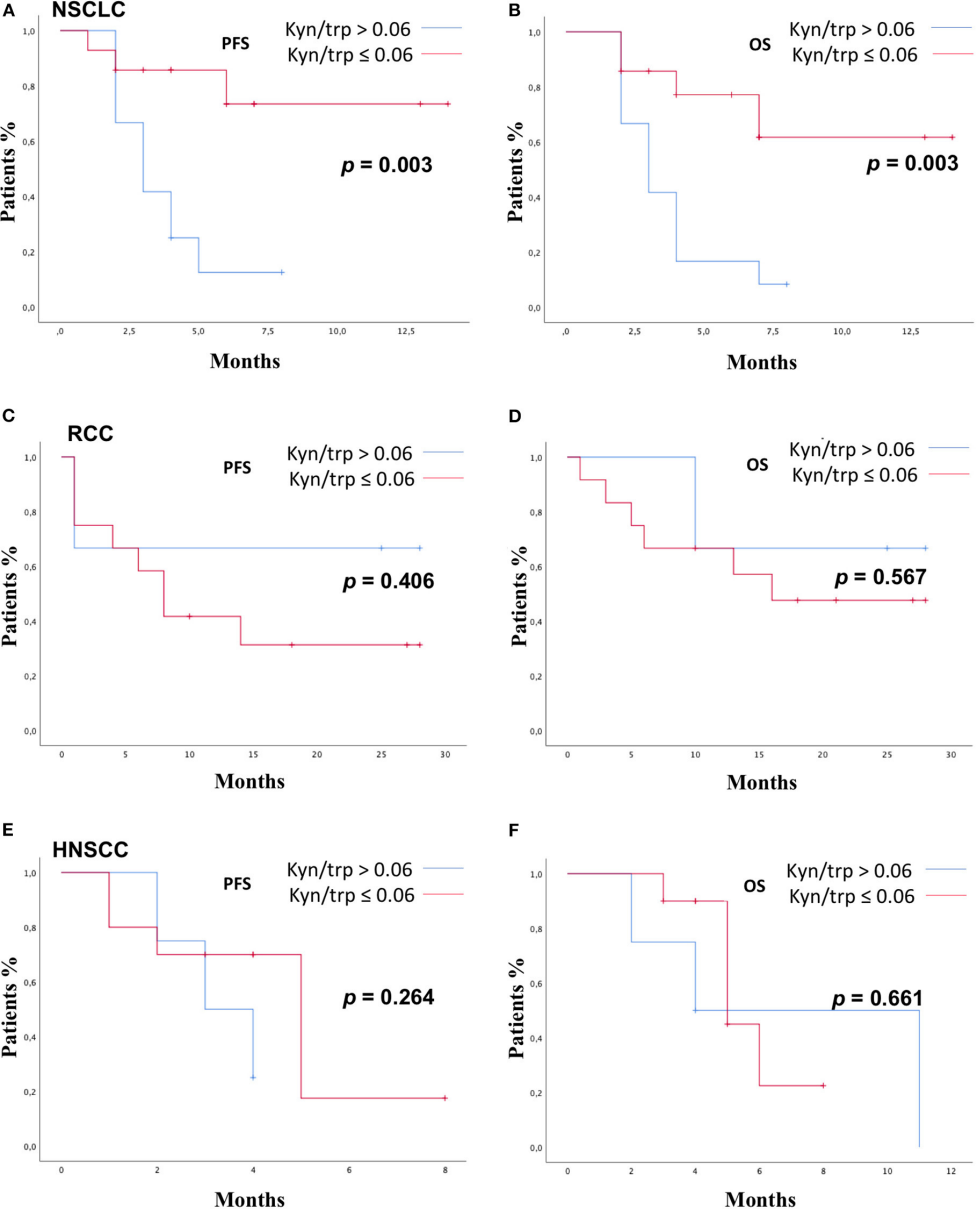
PFS and OS according to kyn/trp in NSCLC group **(A,B)**, RCC group **(C,D)**, and HNSCC group **(E,F)** were addressed by the Kaplan–Meier method and log-rank test. PFS, progression-free survival; OS, overall survival; kyn, kynurenine; trp, tryptophan; NSCLC, non-small cell lung cancer; RCC, renal cell carcinoma; HNSCC, head and neck squamous cell carcinoma.

Patients were stratified by median baseline value of serum kyn/trp (0.06) in patients with kyn/trp ratio >0.06 and ≤0.06. PFS was longer in patients presenting lower values of kyn/trp than in patients showing higher values (median PFS 8 vs. 3 months; HR: 0.49; 95% CI 0.24–1.02; *p* = 0.058). Patients with lower kyn/trp ratio showed even a significantly better OS than did patients with a higher kyn/trp ratio value (median OS 16 vs. median 4 months; HR: 0.39; 95% CI 0.19–0.82; *p* = 0.013) (Figures 3A,B). As it is shown in Figure 4, referring to the primary tumor, only in the NSCLC group (Figures 4A,B) PFS and OS were significantly longer in patients with kyn/trp ratio ≤0.06 vs. kyn/trp >0.06 (median PFS not reached vs. 3 months; range 1.8–4.1; *p* = 0.003 and median OS not reached vs. 3 months, range 1.8–4.1; *p* = 0.003, respectively). Instead, in the RCC group (Figures 4C,D), median PFS was not reached vs. 8 months (range 4.6–11.3; *p* = 0.406), and OS was not reached vs. 16 months (range 11.0–23.6; *p* = 0.567) in patients with low and high kyn/trp ratios, respectively. Overall, in RCC, a lower kyn/trp ratio is inclined to be associated with a worse survival despite the lack of statistical significance.

In the HNSCC group (Figures 4E,F), median PFS was 3 months (range 1.0–4.9) vs. 5.0 months (range 3.2–6.2) in patients with kyn/trp >0.06 and kyn/trp ratio ≤ 0.06, respectively (*p* = 0.264). Median OS was 4.0 months (range 0.0–9.8) in patients with kyn/trp ratio > 0.06 vs. 5.0 months (range 3.3–6.6) in patients with kyn/trp ratio ≤ 0.06 (*p*-value = 0.661).

## Discussion

In our study, including different solid tumors, baseline serum kyn/trp ratio is associated with early progression and survival, confirming its role as a possible predictive biomarker of primary resistance to immunotherapy. In particular, higher baseline kyn/trp value is associated with early progression and, consequently, poor prognosis. This study confirms the previous results in NSCLC cohort of patients (37). As a matter of fact, in our analysis, the statistical significance of the association between kyn/trp ratio and response to immunotherapy is strong when considered in the NSCLC population, whereas it is weak in the overall study population. Moreover, considering RCC and HNSCC separately, there is no correlation between kyn/trp ratio and response, suggesting that in these two types of tumors, the IDO activity could have a marginal role in the complex mechanism determining the primary resistance to immunotherapy or concurrent medications, able to induce TDO expression like the steroids, and nutritional state and infection may have a confounding effects on results. Indeed, the pathway is responsive to unspecific inflammation, and it is induced in chronic immune activation states because IDO is sensitive IFN gamma gene, and it is induced by inflammatory stimuli (27) that may be significant especially in locally relapsed HNSCC, which is generally an inflamed disease. Moreover, the extremely scarce sample size did not allow us to draw definitive conclusions.

In our analysis, kyn/trp ratio resulted to be significantly lower in patients with lung metastases than patients with other metastatic sites. Indeed, immune response could be considered the result of a complex interplay between local tumor microenvironment and peripheral immunity. Moreover, the role of metastatic organ microenvironment in response or resistance to checkpoint inhibitor is still not completely understood. Recently, in a large retrospective study including NSCLC in treatment with immunotherapy, lymph node metastases were associated with the best response, lung and pleura metastases were associated with an intermediate response, and liver, which expressed TDO at high levels, and bone metastases were associated with the least responses to immunotherapy (38). In a study including 102 patients with NSCLC in treatment with nivolumab, lung and liver metastases have been proven to be excellent parameters in predicting OS (39). Consequently, metastatic sites could have an impact on the development of primary resistance to immunotherapy (40), but the underlying biological mechanism should be further investigated, and our study must be considered as a hypothesis generator.

Looking at the baseline patients characteristics, kyn/trp ratio is slightly significantly lower in female compared with male patients. This result could be explained by the sexual dimorphism of the immune system able to influence the response to immunotherapy (41, 42). In a recent meta-analysis (43), the magnitude of benefit from immunotherapy was sex dependent with an improving effectiveness in male patients. However, in our study, there is no evidence that the early progression to immunotherapy is sex dependent, and the correlation between sex and IDO activity should be further evaluated in a large study population.

Considering the different subgroups on the basis of primary tumor site, the association between kyn/trp ratio and early progression was statistically significant in NSCLC, confirming the results from our previous report (37). Moreover, considering the different subgroups based on histology, the association between kyn/trp ratio and early progression was statistically significant in squamous histology group. IDO activity was recently investigated in different solid squamous tumors. In squamous cervical cancer, IDO activity expressed in terms of kyn/trp ratio was shown to be linked to poor survival (43). In squamous esophageal carcinoma, a high tissue IDO expression was associated with impaired OS and aggressive disease (44). Moreover, in a recent study including 88 squamous oral cavity carcinoma, high tissue IDO expression was associated with OS, acquiring the role of negative prognostic factor (45). These results support our data by suggesting that IDO could have a central role in the development of primary resistance to immunotherapy in tumor with squamous histology, regardless of the tumor site. In the RCC and HNSCC subgroups, we failed to demonstrate a significant association between serum kyn/trp ratio and early progression. In particular, in RCC, kyn/trp ratio seems to have a reverse trend because patients with low baseline ratio tend to often experience early progression, although the statistical significance was not reached. The prognostic and predictive values of kyn/trp ratio in the different disease could be influenced by the type of treatment administered in a first-line setting. In our study, all patients with RCC received TKI in the first line. In metastatic RCC, in the phase III study (8) comparing nivolumab with everolimus, a subgroup analysis found that patients previously treated with pazopanib showed statistically significant increase in OS with nivolumab, whereas patients previously treated with sunitinib did not show significant difference in OS between nivolumab and everolimus (8, 46). Thus, previous therapy with TKIs might enhance subsequent immunotherapy efficacy by different mechanisms. As a matter of fact, therapy with TKIs could induce a reduction in Treg levels and MDSCs, improving type 1 cytokine response (47, 48). Moreover, TKIs could also regulate the expression of NK cell ligands in tumor cells, conferring sensitivity to NK cell lysis, and normalize tumor vascularization, allowing helping CD8 T-cell influx into the tumor (49). Despite this immunomodulatory effect, it is still unknown whether and how TKIs could also influence IDO activity. Also, chemotherapy could have an immunomodulatory power. In our study population, patients with HNSCC and NSCLC received platinum-based chemotherapy in the first-line setting. Cisplatin-based regimen enhanced the T-cell activation and proliferation and their cytotoxic activity and inhibited the immunosuppressive pathways (50). Nevertheless, the effect of chemotherapy on IDO activity is unknown and should be further investigated.

In the multivariate analysis, probably the kyn/trp ratio loses statistical significance as a result of the heterogeneity of the resistance mechanisms to immunotherapy involved in the different tumor types. These mechanisms are not yet completely understood and deserved further investigation according to the primary tumor biology and previous treatment.

A possible limitation of the study is due to the presence of possible confounding factors that could interfere with a correct interpretation of serum kyn/trp ratio. First of all, malnutrition could modify the circulating trp levels representing a relevant issue in the management of HNSCC patients. In our study cohort population with HNSCC, nutritional support was provided to all patients with feeding difficulties. Secondly, serum kyn/trp ratio is the result of the activity of some different enzymes including mainly IDO and TDO2 (51). The enzyme TDO2 is expressed in the liver and is involved in the catabolism of trp. However, TDO2 was shown to be overexpressed in some tumor cells as a mean of immune escape (27, 52, 53). Thus, TDO seems to contribute to cancer-associated inflammation and tumor progression like IDO, and it is not possible to distinguish the activity of the two enzymes on the basis of serum ratio.

Nevertheless, measuring serum kyn/trp levels could be a reliable method to evaluate the overall impact of trp depletion in determining primary resistance to immunotherapy. To date, chromatography remains one of the most sensitive and accurate methods for quantifying both trp and kyn from biological matrices and measure trp catabolism.

One more limitation to be acknowledged is a relative small sample size of study population, with potential for inherent biases. Certainly, a prospective validation on a larger sample size is required to assess reproducibility and generalizability of our results.

Recently, the FDA-approved pembrolizumab in pediatric and adult solid tumors with microsatellite instability (MSI) or mismatch repair deficiency led to the first approval based on a specific biomarker rather than the organ-specific histology (54, 55). As well as MSI and tumor mutational burden (TMB), in the future, the enzyme pathways involved in trp catabolism could have the potential to become an additional agnostic biomarker of primary resistance to immunotherapy beyond the histology and tumor site.

Actually, several clinical trials are investigating anti-IDO agents in several solid tumors in monotherapy or in combination strategy with other drugs (Table 4). The majority of IDO inhibitors are direct enzymatic inhibitors, such as epacadostat and navoximod, whereas the trp mimetic indoximod acts directly on immune cells, creating an artificially trp-mediated signal to reverse the IDO-related immunosuppressive mechanism (56).

**Table 4:** Ongoing phase II/III trial evaluating IDO inhibitor or tryptophan mimetic agents in monotherapy or in combination strategy with others drugs.

**Study title**	**Study number**	**Condition or disease**	**Treatments**	**Status—phase**
A phase 2 study of the IDO inhibitor epacadostat vs. tamoxifen for subjects with biochemical-recurrent-only EOC, PPC or FTC following complete remission with first-line chemotherapy	NCT01685255	Ovarian cancer Genitourinary (GU) tumors	Epacadostat Tamoxifen	Terminated—phase 2
A phase II double-blinded, randomized, placebo-controlled study of indoximod in combination with a taxane chemotherapy in metastatic breast cancer	NCT01792050	Metastatic breast cancer	Docetaxel or paclitaxel Indoximod	Accrual completed—phase 2
A phase II trial of IDO-inhibitor, BMS-986205, and PD-1 inhibitor, nivolumab, in patients with recurrent or persistent endometrial cancer or endometrial carcinosarcomas (CA017-056)	NCT04106414	Endometrial adenocarcinoma Endometrial carcinosarcoma	Nivolumab BMS-986205	Recruiting—phase 2
A phase 1/2 study of the concomitant administration of indoximod plus immune checkpoint inhibitors for adult patients with advanced or metastatic melanoma	NCT02073123	Metastatic melanoma Stage III melanoma Stage IV melanoma	Indoximod Ipilimumab Nivolumab Pembrolizumab	Recruiting—phase 1/2
A phase I/II study of the combination of indoximod and temozolomide for adult patients with temozolomide-refractory primary malignant brain tumors	NCT02052648	Glioblastoma multiforme Glioma Gliosarcoma Malignant brain tumor	Indoximod + temozolomide Temozolomide Bevacizumab Stereotactic radiation	Completed—phase 1/2
A phase I/II study of indoximod in combination with gemcitabine and Nab-paclitaxel in patients with metastatic adenocarcinoma of the pancreas	NCT02077881	Metastatic pancreatic adenocarcinoma Metastatic pancreatic cancer	Nab-paclitaxel Gemcitabine Indoximod	Completed—phase 1–2
A phase II study of epacadostat and pembrolizumab in patients with imatinib refractory advanced gastrointestinal stromal tumors	NCT03291054	Gastrointestinal stromal tumors	Pembrolizumab Epacadostat	Recruiting—phase 2
A phase I/IIb study of DEC205mAb-NY-ESO-1 fusion protein (CDX-1401) given with adjuvant poly-ICLC in combination with INCB024360 for patients in remission with epithelial ovarian, fallopian tube, or primary peritoneal carcinoma whose tumors express NY-ESO-1 or LAGE-1 antigen	NCT02166905	Fallopian tube carcinoma Ovarian carcinoma Primary peritoneal carcinoma	Biological: DEC-205/NY-ESO-1 Fusion Protein CDX-1401 Epacadostat	Recruiting—phase 1/2
Phase I/II trial of BMS-986205 and nivolumab as first or second-line therapy in hepatocellular carcinoma	NCT03695250	Metastatic hepatocellular carcinoma Stage III hepatocellular carcinoma AJCC v8 Stage IIIA hepatocellular carcinoma AJCC v8	IDO1 inhibitor BMS-986205 Nivolumab	Recruiting—phase 1/2
A phase II study to determine the safety and efficacy of INCB024360 in patients with myelodysplastic syndromes	NCT01822691	Myelodysplastic syndromes	INCB024360	Terminated—phase 2
Influence of the celecoxib administration before surgery for endometrial cancer on indoleamine 2,3-dioxygenase 1 (IDO1) tumor expression and immune cells tumor's infiltration	NCT03896113	Endometrium cancer	Drug: celecoxib 200-mg capsule	Recruiting—phase 2
Window-of-opportunity trial of nivolumab and BMS986205 in patients with squamous cell carcinoma of the head and neck (CA017-087)	NCT03854032	Lip Oral cavity squamous cell carcinoma Pharynx Larynx squamous cell carcinoma	Nivolumab IDO1 inhibitor BMS-986205	Recruiting—phase 2
Phase II study of epacadostat (INCB024360) in combination with pembrolizumab in patients with locally advanced/metastatic sarcoma	NCT03414229	Locally advanced/Metastatic sarcoma	Epacadostat Pembrolizumab	Active not recruiting—phase 2
A phase II pilot trial of an indoleamine 2,3, dioxygenase-1 (IDO1) inhibitor (INCB024360) plus a multipeptide melanoma vaccine (MELITAC 12.1) in patients with advanced melanoma	NCT01961115	Advanced/metastatic melanoma (mucosal, uveal, skin)	Epacadostat MELITAC 12.1 peptide vaccine	Completed—phase 2
A phase 1/2, open-label, safety, tolerability, and efficacy study of epacadostat in combination with pembrolizumab and chemotherapy in subjects with advanced or metastatic solid tumors (ECHO-207/KEYNOTE-723)	NCT03085914	Solid tumors Colorectal cancer (CRC) Adenocarcinoma (PDAC) Lung cancer UC (urothelial cancer) Head and neck cancer	Epacadostat Pembrolizumab Oxaliplatin 5-Fluorouracil Gemcitabine Nab-paclitaxel Carboplatin Paclitaxel Pemetrexed Cyclophosphamide Cisplatin	Active not recruiting—phase 1–2
A phase 1/2, open-label, dose-escalation, safety, tolerability, and efficacy study of epacadostat and nivolumab in combination with immune therapies in subjects with advanced or metastatic malignancies (ECHO-208)	NCT03347123	Solid tumors	Epacadostat Nivolumab Ipilimumab Lirilumab	Active not recruiting—phase 1–2
A phase 1/2 study of relatlimab (anti-LAG-3 monoclonal antibody) administered in combination with both nivolumab (anti-PD-1 monoclonal antibody) and BMS-986205 (IDO1 inhibitor) or in combination with both nivolumab and ipilimumab (anti-CTLA-4 monoclonal antibody) in advanced malignant tumors	NCT03459222	Advanced cancer	Relatlimab Nivolumab BMS-986205 Ipilimumab	Recruiting—phase 1–2

The phase I/II KeyNote 037/ECHO 301 trial (57) evaluated epacadostat at different dose levels and pembrolizumab, an anti-PD-1 agent 200 mg every 3 weeks, in 62 patients with advanced solid tumors, showing promising results. High-grade toxicities occurred in 24% of patients, and no adverse events led to death. Objective response was achieved in 12 out of 22 patients with several types of solid tumors.

Moreover, the phase II trial (58), evaluating indoximod and pembrolizumab in naïve patients for immunotherapy with advanced melanoma, showed promising results with an objective response rate of 55.7% and a median PFS of 12.4 months.

Unfortunately, the phase III trial KeyNote/ECHO 301 (59), evaluating epacadostat 100 mg twice daily (BID) and pembrolizumab 200 mg every 3 weeks in naïve patients for immunotherapy with metastatic melanoma, failed to meet its primary endpoint of improving PFS.

To maximize the benefit of these combination strategies, it is important to improve the selection of clinical trial population through the detection of serum and/or tissue biomarkers.

Therefore, the role of serum kyn/trp should be evaluated and interpreted in the contest of other circulating and tissue immunological parameters: different T-cell subpopulations, MDSCs, circulating cytokines and chemokines, the immune cell death biomarkers, and other possible predictive biomarkers, such as T-cell immunoglobulin mucin-3 (TIM3), lymphocyte-activation gene-3 (LAG3), and T-cell immunoglobulin and ITIM domain (TIGIT) (60, 61). In our study, serum kyn/trp has been confirmed to be a possible prognostic and predictive biomarker of primary resistance to immunotherapy in patients with solid tumors in treatment with immunotherapy, regardless of the primary tumor histology, although its relative weight is significantly related to gender, site of metastasis, lung cancer, and squamous histology. However, the impact of serum kyn/trp levels on prognosis and resistance to immunotherapy should be further investigated and its role integrated together with other possible and dynamic mechanisms of resistance to immunotherapy treatment and definitively validated in further studies.

## Data Availability Statement

The datasets generated for this study are available on request to the corresponding author.

## Ethics Statement

This study was approved by local ethics committee of Sapienza University of Rome, RIF. CE: 4421. The patients/participants provided their written informed consent to participate in this study.

## Author Contributions

AB and LL conceived the study. AB, MN, and PM designed the work. AB, SM, GP, and BC wrote the manuscript. LL, MR, RG, AC, EC, IZ, and SS acquired the samples, performed experiments, and acquired data. AB and MR analysed the data. AB, MN, PM, SM, MS, and GP discussed the results and implications of findings. All authors contributed to the article and approved the submitted version.

## Conflict of Interest

PM has/had a consultant/advisory role for BMS, Roche, Genentech, MSD, Novartis, Amgen, Merck Serono, Pierre Fabre, and Incyte. The remaining authors declare that the research was conducted in absence of any commercial or financial relationship that could be construed as a potential conflict of interest.
